# Interaction between adipocytes and high-density lipoprotein:new insights into the mechanism of obesity-induced dyslipidemia and atherosclerosis

**DOI:** 10.1186/s12944-019-1170-9

**Published:** 2019-12-16

**Authors:** Tianhua Zhang, Jin Chen, Xiaoyu Tang, Qin Luo, Danyan Xu, Bilian Yu

**Affiliations:** Department of Cardiovascular Medicine, the Second Xiangya Hospital, Central South University, Changsha, Hunan 410011 People’s Republic of China

**Keywords:** Adipocyte, Cholesterol, HDL, Cardiovascular disease, Obesity

## Abstract

Obesity is the most common nutritional disorder worldwide and is associated with dyslipidemia and atherosclerotic cardiovascular disease. The hallmark of dyslipidemia in obesity is low high density lipoprotein (HDL) cholesterol (HDL-C) levels. Moreover, the quality of HDL is also changed in the obese setting. However, there are still some disputes on the explanations for this phenomenon. There is increasing evidence that adipose tissue, as an energy storage tissue, participates in several metabolism activities, such as hormone secretion and cholesterol efflux. It can influence overall reverse cholesterol transport and plasma HDL-C level. In obesity individuals, the changes in morphology and function of adipose tissue affect plasma HDL-C levels and HDL function, thus, adipose tissue should be the main target for the treatment of HDL metabolism in obesity. In this review, we will summarize the cross-talk between adipocytes and HDL related to cardiovascular disease and focus on the new insights of the potential mechanism underlying obesity and HDL dysfunction.

## Introduction

Obesity is the most common nutritional disorder worldwide and is one of the major risk factors for atherosclerotic cardiovascular disease (ASCVD) [1]. One of the proatherogenic effects of obesity is attributable to its accompanying dyslipidemia. The prominent dyslipidemia in obesity is low high density lipoprotein (HDL) cholesterol (HDL-C) levels and apolipoprotein A-I (apoA-I). Epidemiological studies have shown a strong inverse correlation between HDL-C, apoA-I and obesity [2, 3]. Specifically, the correlation of low HDL-C with obesity is strongest with central obesity which is characterized by visceral fat deposition [4, 5]. In addition to low HDL-C level, under the state of obesity, cholesterol efflux capacity, nitric oxide-mediated endothelial function, which are the main metrics of HDL function, are impaired in obese patients or *ob/ob* mice [6–8]. It is widely known that low HDL-C level is part of the atherogenic dyslipidemia and is strongly and inversely correlated with ASCVD risk. Most importantly, a large number of recent studies have highlighted the possibility that HDL associated metrics other than HDL-C, such as HDL particle concentration, HDL composition, and HDL function are more likely determinants of ASCVD risk [9]. Thus, the mechanisms underlying obesity and HDL alterations are of considerable importance. The overproduction of free fatty acid (FFA) and very low density lipoprotein (VLDL) is regarded as one of the reasons responsible for low HDL-C level [10]. What’s more, some key enzymes involved in HDL metabolism, such as cholesteryl ester transfer protein (CETP), lecithin/cholesterol acyltransferase (LCAT), hepatic lipase (HL) and protein phospholipid transfer protein(PLTP), are changed in people with obesity who have insulin resistance [11]. In spite of these interpretations, the mechanisms for the association of obesity and HDL remain to be fully elucidated.

As we know, adipose tissue is a metabolically dynamic organ, serving as a buffer to control energy metabolism and a regulator of endocrine function related to cardiovascular disease. Abnormal excess energy in obesity leads to hypertrophied adipocytes, which manifest several altered metabolic properties that could play a key role in the obesity-related HDL alterations. Furthermore, recent studies suggested that HDL had reciprocal effect on obesity. In this review, we will focus on the updated understanding of the interaction between adipocytes and HDL, to provide new clues to help us understand the potential mechanism underlying obesity, HDL and atherosclerosis.

## HDL metabolism and function, and its relationship with ASCVD

Reverse cholesterol transport (RCT) is generally thought to be the central antiatherogenic effect of HDL [12]. In the process of RCT, HDL can mediate the removal of cholesterol from peripheral cells and deliver it to the liver for excretion. The first step involves secretion of apoA-I, mainly by the liver and the intestine. Secreted apoA-I interacts functionally with ATP-binding cassette sub-family A member 1 (ABCA1), and this interaction leads to the transfer of cellular phospholipids and cholesterol to lipid-poor apoA-I. The lipidated apoA-I is then gradually converted to disc-shaped particles enriched in unesterified cholesterol. The esterification of free cholesterol by the enzyme LCAT converts the disc-shaped particles to spherical HDL particles, which can further promote cellular cholesterol efflux through ATP-binding cassette transporter G1 (ABCG1) and scavenger receptor class B type I (SR-BI) [13, 14]. As HDL particles get more cholesterol, they engage in the exchange with triglycerides-rich lipoproteins mediated by cholesterol ester transfer protein (CETP). The remaining HDL particles return to the liver, interacting with SR-BI receptor which removes cholesterol for bile acid excretion [15, 16]. In addition to RCT, HDL also possess several other putative atheroprotective functions, associated to the anti-inflammatory, anti-thrombotic and anti-oxidant properties as well as to the ability to support endothelial physiology [17, 18].

To date, many clinical studies have shown that HDL-C is negatively associated with the risk of ASCVD [19–22]. For a 5 mg/dl decrement in HDL-C level, there was a 14% incremental risk of ASCVD events [23]; in contrast, each 1 mg/dl increase in HDL-C level is associated with a 2–3% reduction in the risk of ASCVD [24]. However, neither altering HDL-C levels genetically nor raising HDL-C with certain drugs that elevate HDL-C, such as niacin and CETP inhibitors, translates into beneficial cardiovascular outcomes [25–30].

As we know, in human plasma, HDL is a heterogeneous collection of lipoprotein particles ranging in diameter from 7 to 12 nm, and with a density from 1.063 to 1.21 g/ml [31]. HDL particles of different sizes contain different amount of cholesterol, and the largest HDL particles contain the most cholesterol [32]. Thus, HDL-C levels do not necessarily correlate with HDL particle (HDL-P) concentration, and the increase in HDL-C level is most likely a reflection of a relative increase in HDL particle size [9, 33]. Furthermore, HDL-C levels are influenced by many different variables that also affect CVD risk, such as sex, smoking, alcohol, diet, aerobic exercise, obesity, type 2 diabetes, and systemic inflammation [34–38]. Whereas, HDL-P levels appear to be correlated to a lesser extent with these factors [39]. Thus, there is growing recognition that cardiovascular risk was better reflected by numbers of lipoprotein particles than by the amount of cholesterol these particles contained [39–41].

Because of the dissociation between improvements in HDL function and HDL-C levels [42, 43], more researches concern the link between HDL function and the incidence of cardiovascular disease. It has been shown that cholesterol efflux capacity, a biomarker that characterizes a key step in RCT, was inversely associated with the incidence of cardiovascular events in a population-based cohort [44]. Many other changes in HDL function, ranging from antioxidant potential to the ability to regulate nitric oxide synthase activity in endothelial cells, have been proposed to be clinically relevant [45]. Taken together, HDL-associated metrics other than HDL-C, such as HDL-P concentration and HDL function, could be more likely determinants of ASCVD risk.

## Adipose tissue involved in energy and secretion metabolism related to ASCVD

### Adipose tissue types and their roles in energy metabolism

Adipose tissue is the main site for energy storage and is found throughout the body in distinct subcutaneous and visceral depots [46]. According to its morphology, location, and function, adipose tissue can be broadly classified into two main types: white adipose tissue (WAT) and brown adipose tissue (BAT).

In humans, WAT is dispersed throughout the body, with major intra-abdominal depots around the omentum, intestines, and perirenal areas, as well as in subcutaneous depots in the buttocks, thighs, and abdomen [47]. Mature white fat cells contain a single large droplet. The lipid droplets contain a mixture of neutral fats, triglycerides, fatty acids, phospholipids, and cholesterol, in which triglycerides comprise approximately 95% [48]. As an energy-supplying organ, WAT can release energy through the sequential hydrolysis of triglycerides stored in cell lipid droplets [49]. Apart from storing triglycerides, WAT is also the body’s largest free cholesterol reservoir [50]. It contains 1–2 mg of cholesterol per gram of wet weight, majority being in the free, unesterified form. In obesity, over half of the total body cholesterol may reside within this tissue [50]. However, WAT is not only a site of cholesterol storage. Recent studies reported that the cholesterol in WAT could efflux to HDL [51], meaning that WAT is also involved in overall cholesterol metabolism.

WAT is not simply an inert storage depot of lipids, but is also an important endocrine organ. It can secrete a number of protein factors that are collectively called adipokines. Adipokines are involved in the regulation of energy intake and balance, in blood pressure regulation, in vascular hemostasis, in lipid metabolism, in carbohydrate homeostasis, and in angiogenesis, as well as growth factors and active phase and stress response proteins. Adipokines are also related to the immune system, including classical cytokines, such as tumor necrosis factor-α (TNF-α), interleukin 6, and interleukin 8 [52].

BAT, in human fetuses and newborns, is found in axillary, cervical, perirenal, and periadrenal regions, but decreases shortly after birth and has traditionally been considered insignificant in adults [47, 53, 54]. Brown adipocytes are smaller than white adipocytes, their cytoplasm contains several lipid droplets, a roundish nucleus and numerous, large, generally spherical mitochondria with laminar cristae [53]. The multilocality of BAT maximizes the cytoplasmic-lipid interface, making large amounts of fatty acids available quickly for mitochondrial uncoupling and consequent thermogenesis [54]. BAT is an important player in energy expenditure because of its ability to convert energy toward heat using uncoupling protein 1 (UCP1), a process called non-shivering thermogenesis [55]. The high metabolic activity of BAT and adipose tissue browning, referring to the formation of so-called beige adipocytes in WAT [56, 57], suggest that the activation of brown and beige adipocytes may be successfully targeted to combat metabolic and cardiovascular diseases in humans.

Recent research also found evidence of the existence of BAT-derived endocrine factors using BAT transplantation, which led to a significant reduction in body weight gain with increased oxygen consumption and decreased total body fat mass by activating endogenous BAT, resulting in improvement of insulin resistance and liver steatosis [58, 59]. Acting as a secretory organ, BAT can also release active thyroid hormone triiodothyronine [60], and produces fibroblast growth factor 21 after thermogenic activation [61]. Retinol binding protein-4 is also produced and regulated by BAT [62].

### Dysfunctional adipose tissue and atherosclerosis

In the state of obesity, white adipocyte dysfunction occurs when excess circulating fatty acids flux into adipose tissue exceeds the ability of adipocytes to store the excess energy as triglycerides. Dysfunctional white adipocytes release more fatty acids due to increased basal lipolysis and resistance to insulin. Lipolysis and the subsequent rise in circulating free fatty acid lead to the development of hypertriglyceridemia and insulin resistance in peripheral organs [46]. Additionally, free fatty acid are actively involved in modulating several signalling pathways that mediate inflammation in cells involved in atherosclerosis development [63]. Besides releasing fatty acids, white adipocytes dysfunction is associated with upregulation of inflammatory and apoptotic signalling pathways, endoplasmic reticulum (ER) stress, increased release of pro-inflammatory adipokines and chemokines [64]. In contrast, some adipokines, such as adiponectin and omentin, which have a protective role in atherosclerosis development, are reduced with increased adiposity [65, 66]. Thus, by inducing dyslipidemia and inflammation, secreting elevated levels of pro-inflammatory adipokines or reduced levels of anti-inflammatory adipokines, WAT participates adversely in the progression of atherosclerosis development in obesity [67, 68].

Unlike WAT, BAT has been identified as a key player in triglyceride clearance because thermogenesis of BAT consumes large amounts of fatty acids [69]. Furthermore, BAT activation decreases cholesterol levels as well [70–72]. Thus, BAT is increasingly recognized as a potential therapeutic target to combat atherosclerosis development [73, 74]. Indeed, it has been shown that activating BAT by β3-adrenergic receptor stimulation exhibits a protective role from atherosclerosis development in mice [72]. Vice versa, BAT dysfunction generated by knocking out the insulin receptor in BAT specifically in ApoE−/− mice aggravates the atherosclerosis process, characterized by a significant increase of lipid depots, atherosclerotic coverage, lesion size and complexity, increased macrophage infiltration and proinflammatory markers expression [75]. As obesity is a state of low thermogenic adipocyte activity, evidenced by lipid accumulation and mitochondrial dysfunction and loss, i.e.“whitening” [76–79], future research might focus on the potential to activate BAT in obese individuals, despite its whitened phenotype.

## The HDL: adipocyte connection

Although macrophage-specific RCT is thought to be the most important mechanism that HDL exerts cardioprotective effects, macrophages have minimal contribution to total cholesterol content in plasma and they don’t regulate circulating HDL-C levels [80]. Nowadays, there is increasing evidence that adipose tissue has a potential contribution to HDL metabolism. Considering the large area of fat depot, it is tempting to speculate that adipose tissue exerts a great contribution to HDL-C levels.

### The cholesterol exchange between adipose tissue and HDL particles: implying a role of adipocytes in HDL-C level

Adipose tissue is the body’s largest cholesterol pool. A particular characteristic of adipocytes is that cholesterol is found mostly in its free, non-esterified form because of lack of acyl-coenzyme A: cholesterol acetyltransferase (ACAT) enzyme activity [81]. This clearly distinguishes the adipocyte from other cell types such as steroid-hormone-producing adrenal cells or cholesterol-laden foam cells of atherosclerotic lesions, which have the capacity to accumulate considerable quantities of excess cholesteryl esters (CE) [82]. Previously, adipocytes were considered as the passive cholesterol-buffering sink. In fact, excess free cholesterol is deleterious to cells [83]. Therefore, it is likely that free cholesterol accumulated in adipocytes would export to circulating lipoproteins to maintain cellular cholesterol homeostasis. A decrease in intracellular cholesterol content was associated with an increase in serum lipoprotein cholesterol levels in experimental animals under certain fasting conditions [84–86]. Conversely, diet-induced hypercholesterolemia can lead to an elevation of the adipocyte cholesterol level [50, 87, 88]. These observations suggested that a dynamic equilibrium might exist between serum lipoprotein cholesterol and adipocyte cholesterol pools. Subsequent studies have shown that adipose tissue expresses high levels of key cholesterol transporters ABCA1 and SR-BI [89, 90], providing a gateway for cholesterol to efflux onto apoA-I or HDL particles. Prattes et al. [91] have demonstrated that extracellular cholesterol acceptors β-cyclodextrin or apoA-I can induce energy-dependent intracellular cholesterol trafficking between lipid droplet and plasma membrane (PM), and then remove cholesterol from PM, raising the possibility that adipocytes have the ability to efflux cholesterol. Based on the above in vitro study, Zhang et al. [51] injected adipocytes labeled with ^3^H-cholesterol into the peritoneal cavity of mice to track ^3^H-cholesterol movement and confirmed that adipocytes are a regulated source of cholesterol transfer to HDL both in vitro and in vivo. In contrast to liver and macrophages, adipocyte cholesterol efflux is controlled by ABCA1 and SR-BI, but not ABCG1.

However, the study was inherently limited in its capacity to accurately determine the amount of cholesterol that was transported to HDL and the potential impact on plasma HDL-C levels. Subsequently, Chung et al. [92] determined the contribution of adipose tissue ABCA1 to HDL biogenesis in vivo by using adipocyte-specific ABCA1 knockout mice. Deletion of ABCA1 in adipocytes resulted in their failure to efflux cholesterol to apoA-I and a significant decrease in plasma HDL cholesterol (~ 15%) and apoA-I (~ 13%) concentrations. Little difference in plasma decay of ^125^I-HDL tracer was evident between genotypes, indicating that the reduction of HDL-C was attributable to impaired HDL biogenesis and not to increased clearance. Collectively, these results above establish a novel role for adipocyte in whole-body cholesterol balance and in vivo HDL production.

As a consequence of the low activity in cholesterol synthetic pathway, the majority of adipocyte cholesterol originates from circulating lipoproteins. Thus, in addition to cholesterol efflux from adipocytes to HDL, adipocytes have also developed a dual mechanism to extract cholesterol from HDL in the circulation; one is based on the typical HDL receptor SR-BI, the other is based on a novel “efflux recapture” process that needs apolipoprotein E and the LDL receptor related protein (LRP) [93–95].

Taken together, through the uptake and efflux pathway, adipocytes establish a communication between the adipose tissue-free cholesterol depot and the blood cholesterol pool, which is in favor of cholesterol homeostasis in general, as well as in the adipocyte. Because adipocyte cholesterol accumulation is associated with triglyceride accretion during adipocyte hypertrophy [96], thus, it is conceivable that impaired adipocyte cholesterol efflux or influx which results in cholesterol retention could damage adipose function. However, a recent study conducted by Cuffe et al. [97] demonstrated that although deletion of ABCA1 in adipocytes results in accumulation of cholesterol within adipose, it can afford adipocytes a protective mechanism to avoid excessive triglycerides accumulation. The reason for the discrepancy may be attribute to the high levels of plasma membrane cholesterol in ABCA1 knockout adipocytes. In contrast, adipocyte cholesterol moves from the plasma membrane to the lipid droplet during hypertrophy, leading to enlarged adipocytes and relative depletion of plasma membrane cholesterol [98].

### Adipose tissue can influence HDL-C level by lipolysis

In addition to cholesterol exchange, WAT lipolysis can also modulate HDL-C levels. Several small cohort studies used different hormones [99–102], such as catecholamines and insulin, to stimulate or inhibit adipocyte lipolysis in patients with familial combined hyperlipidemia and endogenous hypertriglyceridemia to detect changes in serum lipids. These studies showed that there is an association between the HDL-C level and lipolysis. However, in these studies, the subjects were patients with metabolic disorders; therefore, the stimulatory or inhibitory effect by hormones would be attenuated by related changes caused by these disorders. Recently, Ryden et al. [103] performed an experiment on 1066 healthy women and men with no other metabolic diseases to examine spontaneous lipolysis and the effects of the major hormones that stimulate and inhibit lipolysis. They found that resistance to the antilipolytic effect of insulin and a high rate of basal lipolysis are associated with low HDL-C and high triglycerides levels. This study further confirmed that lipolysis in subcutaneous fat cells is an important and independent contributor to variations in plasma triglycerides and HDL-C levels. In addition to subcutaneous fat cells, an inverse relationship was also observed between catecholamine-stimulated visceral fat cell lipolysis and HDL-C level [104]. Although the lipolytic activity is higher in visceral than the subcutaneous depot [105], subcutaneous WAT, which is the body’s largest fat depot, is likely to be a much more important contributor to HDL-C level than visceral fat.

In terms of mechanism, increased lipolysis can affect HDL-C level by both direct and indirect effects. On the one hand, WAT lipolysis can hydrolysis triglycerides and release free fatty acids (FFAs) into the plasma, which can be used for hepatic VLDL assembly and secretion. These triglyceride-rich VLDL particles may reduce HDL-C levels by increasing the transfer of triglycerides to HDL, which in turn are hydrolyzed by hepatic lipase. On the other hand, FFAs could induce insulin resistance [106] and alter CETP activity [107], which have roles inHDL metabolism.

### Adipocytes can influence the function of HDL

Recently, emerging evidence indicates that HDL function other than HDL-C, especially cholesterol efflux and RCT, is a stronger predictor of ASCVD risk. Induction of obesity impairs overall RCT in ob/ob mice raising the possibility that adipocytes can influence the function of HDL [8]. Repeated cryostimulation in humans has been described to lower plasma TG while increasing plasma HDL cholesterol levels; and the expression of UCP1 in human epicardial fat is associated with lower plasma TG and higher plasma HDL-cholesterol levels. Furthermore, it has been shown that the activation of thermogenic adipocytes by β3-adrenergic receptor stimulation reduces cholesterol levels and protects from atherosclerosis in transgenic mice expressing both a loss-of-function variant of human apolipoprotein E (APOE*3-Leiden; E3L) and the human CETP (E3L.CETP mice) [72]. These results strongly indicate a role of thermogenic adipocytes in the anti-atherogenic properties of HDL. Zvintzou E et al. [108] showed that APOC3 modulates HDL structure and function, while it selectively promotes BAT metabolic activation. In addition, a recent study conducted by Bartel et al. [77] painstakingly determined that both cold-induced and pharmacological thermogenic adipocytes activation enhance HDL function, which is defined as increased HDL-cholesterol clearance and macrophage-to-faeces RCT. Mechanistically, the authors showed that intravascular lipolysis by adipocyte lipoprotein lipase and hepatic uptake of HDL by SR-BI are the driving forces of HDL-cholesterol disposal in liver. However, further cholesterol efflux experiments from brown and beige adipocytes will be needed to show whether the high lipolytic activity of thermogenic adipocytes not only increases intravascular HDL remodelling and turnover as shown in the above study, but also enhances cholesterol efflux from cells to HDL particles.

### The reciprocal effect of HDL/ apoA-I on adipocyte function

Human adipocytes possess HDL binding sites, which are specific for apoA-I and/or apoA-II, and independent of apoE raising the possibility that HDL might exert reciprocal effect on adipocyte function. Indeed, Van Linthout et al. [109] increased HDL in vivo by human apoA-I gene transfer and supplemented HDL in vitro on partially differentiated adipocytes and subsequently demonstrated that HDL elevates plasma adiponectin concentrations in vivo and increases adiponectin expression in adipocytes in a phosphatidylinositol-3-kinase dependent manner. As adiponectin is an anti-inflammatory adipokine [110], it is likely that the effects of HDL on adiponectin expression may contribute to its anti-inflammatory effects. Recently, Umemoto et al. [111] showed that apoA-I and HDL strongly inhibited palmitate-induced chemotactic factor expression in adipocytes in vitro and that overexpression of human apoA-I led to a reduction of inflammatory gene expression and macrophage accumulation in adipose tissue in mice fed a high-fat diet. These findings further provide evidence that HDL and apoA-I have anti-inflammatory effects on adipocytes and adipose tissue, similar to their better-known effects on vascular cells such as macrophages and endothelial cells. In terms of the mechanism, the anti-inflammatory properties of HDL on adipocytes are in association with disruption and removal of cholesterol from lipid rafts, which are regulated by cholesterol transporters such as ABCA1, ABCG1 and SR-BI [102]. Serum amyloid A (SAA)-containing dysfunctional HDL has been shown to have reduced ability to facilitate cholesterol efflux [112, 113] and thus have loss of the anti-inflammatory properties on adipocytes [114]. However, mechanisms independent of cholesterol efflux to explain these effects of apoA-I and HDL needs to be further determined.

The action of HDL on adipocytes is not limited to cholesterol extraction and anti-inflammatory effects. Considering that HDL favors the release of adiponectin [111], which can increase insulin sensitivity [115], it is of considerable interest to speculate that HDL could therefore exert another beneficial effect bymodulating glucose metabolism. Indeed, in a randomized, crossover, double-blind, placebo-controlled study in patients with type 2 diabetes mellitus, reconstituted HDL treatment reduces plasma glucose levels [116]. In terms of the mechanism, it has been reported that apoA-I can be endocytosed into C2C12 myocytes through a clathrin-dependent endocytotic process, and a global deletion of apoA-I in mice reduces AMP-activated protein kinase (AMPK) phosphorylation and glucose uptake in skeletal muscle, leading to increased fat content and compromised glucose tolerance [117, 118]. In addition to myocytes, increased glucose uptake is also observed in 3 T3-L1 adipocytes after HDL incubation [119]. Thus, it seems reasonable that HDL modulates glucose metabolism through increasing the ability of the fat and muscle to metabolize glucose.

Furthermore, apoA-I and HDL also contribute to modulating body fat content by controlling the extent of lipolysis via hormone-sensitive lipase phosphorylation (p-HSL) [120]. It has also been demonstrated that apoA-I gene overexpression and D-4F treatment lead to significantly increased UCP1 expression in brown adipose tissue and energy expenditure [118]. It is well known that increased expression of UCP1 in brown adipocyte or ectopic expression of UCP1 in mouse or human skeletal muscle and white adipocyte promotes fatty acid oxidation and resistance to obesity. Together, these findings suggest that apoA-I and HDL might have a direct role in the regulation of body weight and are potential pharmacological targets for the treatment of obesity. However, the mechanism underlying the anti-obesity effect of apoA-I remains to be further elucidated.

### The connection of HDL and adipocytes: a tight interconnected link between obesity and HDL metabolic disturbance

Since adipocytes control the levels of HDL as discussed above, why is HDL-C not elevated with obesity because of increased adipose mass and adipose cholesterol in an obese? As we know, obesity is accompanied by a state of chronic, low-grade adipose tissue inflammation. The cross-talk between adipocytes and macrophages accumulated in adipose amplifies the production of pro-inflammatory cytokines and chemokines by macrophages and adipocytes, leading to insulin resistance and an increased risk of cardiovascular disease [121–124]. A fascinating observation is that inflammatory mediators such as TNFα impair cholesterol efflux from adipocytes to HDL [125, 126]. In addition to pro-inflammatory adipokines, reduced levels of anti-inflammatory adipokine adiponectin might also contribute to lower HDL-C levels in the state of obesity. Adiponectin is a collagen-like protein that is exclusively synthesized in WAT. It is induced during adipocyte differentiation, and circulates at relatively high concentrations in serum [127]. Cross-sectional studies have reported that adiponectin is positively associated with HDL-C and negatively associated with triglycerides, which implied an important role of adiponectin in HDL metabolism [128, 129]. Subsequently, Oku et al. [130] confirmed the relationship between adiponectin and HDL-C levels by using adiponectin knockout mice. Notably, when focusing on the mechanism by which adiponectin regulates the level of HDL-C, researchers found that adiponectin can affect ABCA1 expression and apoA-I synthesis in the liver [102, 103], which are essential for HDL assembly. Taking into account adipose inflammation is a hallmark of obesity, it is intriguing to speculate that attenuation of adipocyte-mediated HDL-lipidation and assembly by elevated levels of pro-inflammatory adipokines or reduced levels of anti-inflammatory adipokine adiponectin may directly contribute to lower plasma HDL-C levels in the state of obesity. However, our laboratory observed that the direct effects of inflammation on the cholesterol efflux capacity of adipocytes highly dependent on cytokine concentration [126]. Low levels of TNF-α could increase adipocyte cholesterol efflux within a certain dose range; whereas high concentrations decreased cholesterol efflux from adipocytes. Thus, the notion that inflammation modulate the efflux capacity of adipose tissue and is responsible for the low levels of HDL-C in obesity is somewhat inconclusive and should be further confirmed in obese mice and humans.

It is well-established that the adipose tissue and liver closely coordinate to regulate energy homeostasis in the body through the secretion of metabolically active hormones or metabolites, including fibroblast growth factor 21 (FGF21), adiponectin, uridine, etc. [131, 132]. Because of chronic excessive caloric intake and lack of physical activity,the adipogenic capacity within adipose tissue may falter, which leads to overly hypertrophied adipocytes [133]. The hypertrophic adipocytes release high levels of FFA and its metabolites, which could result in ectopic fat deposition and non-alcoholic fatty liver disease (NAFLD) [134]. Moreover, in the context of obesity, chronic inflammatory state induced by macrophage accumulation in adipose tissue is also associated with aggravated hepatic fibroinflammatory lesions [135, 136]. In addition to adipose, hepatic ABCA1 is critical in maintaining the circulating HDL-C levels by formation of nascent HDL particles [137]. In the state of NAFLD, hepatic free cholesterol content was significantly increased accompanied by decreased levels of ABCA1 and ABCG1 protein [138]. Hence, it can be speculated that energy exchange between adipose tissue and liver could be another possible mechanism underlying the low HDL-C levels in obesity.

**Fig. 1:**
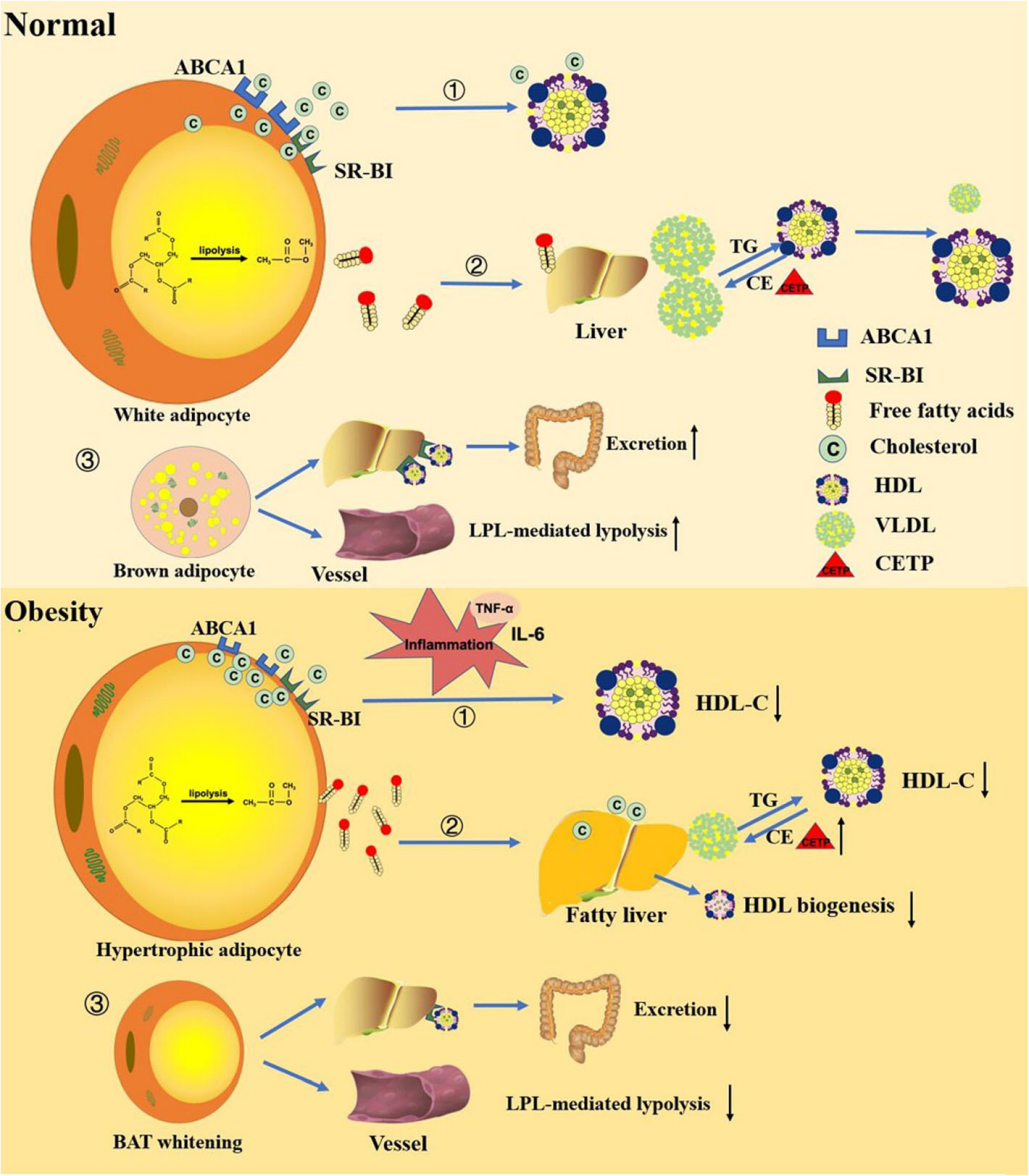
Under normal condition, adipocytes regulate HDL-C levels and HDL function in three ways. ①Intracellular cholesterol can efflux to HDL through ABCA1 and SR-BI receptors on the surface of cell membrane to increase HDL-C levels. ② The free fatty acids released by intracellular triglyceride hydrolysis are used for hepatic production of VLDL, which in turn may reduce HDL-C levels by increasing the transfer of triglycerides to HDL. ③ Brown adipocytes mediate HDL-cholesterol clearance and increase macrophage-to-faeces reverse cholesterol transport through intravascular lipolysis by adipocyte lipoprotein lipase and hepatic uptake of HDL by SR-BI. Chronic inflammation, which is the hallmark of obesity, can impair the cholesterol efflux ability of adipocytes, thereby decrease HDL-C levels in plasma. In addition, enhanced lipolysis of adipocytes under the state of obesity releases more free fatty acids into the blood, leading to hypertriglyceridemia and reduced HDL-C levels. FFAs could also induce insulin resistance and alter CETP activity, which have roles in HDL metabolism. The reverse cholesterol transport capacity of HDL might be impaired because of BAT whitening in obesity

Obesity not only affects the levels of HDL-C in plasma, but also has an impact on the functionality of HDL. However, there is no consolidated explanation for the association between obesity and impaired HDL function. Previous study has shown that obesity was associated with decreases in large HDL subclass distribution [139] and impaired conversion of pre-β1 to pre-β2 HDL [140], which might have some effects on HDL function. In light of recent studies demonstrating the potential of BAT activation in HDL function as mentioned above, and obesity is a state of low thermogenic adipocyte activity [77], it is tempting to speculate that stimulating thermogenic adipocyte activity may protect from cardiovascular disease not only by decreasing remnant lipoproteins levels but also by stimulating HDL-cholesterol flux from atherogenic lesions to the liver for ultimate cholesterol excretion (Fig. 1).

## Conclusions

In conclusion, low level of HDL-C and impaired HDL function are common lipid disorders in the state of obesity and are considered to play an indispensible role in the development of ASCVD. Considering that adipocytes control the levels and function of HDL, it is intriguing to speculate that the connection between HDL and adipocytes represents the potential mechanism underlying HDL metabolic disturbance in obesity. In normal body weight, adipose tissue can maintain normal cardiovascular function by regulating HDL-C levels and HDL function; while in obese individuals, adipose tissue morphology has been changed, which leading to impaired regulatory function and eventually accelerate the occurrence of cardiovascular disease.

However, there are some questions still need to be further explored. First, the distribution of cholesterol in humans and rodents is different [62, 63, 65, 107], as most of the cholesterol in rodents is carried by HDL particles, whereas most of the cholesterol in human is carried by LDL particles. Thus, the results of the experiments in mice may not be applicable to humans. Second, different WAT locations have different metabolic and endocrine characteristics [109, 141]. For example, WAT in the breasts and buttocks is highly sensitive to estrogens, while WAT in the upper back and neck is more sensitive to glucocorticoids. Visceral (intra-abdominal) WAT has an adipokine secretion profile related to inflammation and type 2 diabetes, while subcutaneous WAT shows lower amounts of secretion of proinflammatory adipokines [22]**.** Thus, the results of experiments on tissues from different locations probably should not be compared. In addition, most studies were based on WAT as it is common and easy to obtain; however, the relationship between BAT and HDL needs to be further explored.

## Data Availability

Not applicable.

## Abbreviations

ABCA1: ATP-binding cassette sub-family A member 1
ABCG1: ATP-binding cassette transporter G 1
ACAT: Acyl-coenzyme A: cholesterol acetyltransferase
apoA-I: Apolipoprotein A-I
APOE*3-Leiden; E3L: A loss-of-function variant of human apolipoprotein E mice
ASCVD: Atherosclerotic Cardiovascular disease
BAT: Brown adipose tissue
CE: Cholesteryl esters
CETP: Cholesterol ester transfer protein
E3L.CETP: A loss-of-function variant of human CETP mice
ER: Endoplasmic reticulum
FFA: Free fatty acid
FFAs: Free fatty acids
FGF21: fibroblast growth factor 21
HDL: High-density lipoprotein
HDL-C: HDL cholesterol
HDL-P: HDL particle
HL: Hepatic lipase
LCAT: Lecithin/cholesterol acyltransferase
LDL: Low density lipoprotein
LRP: LDL receptor related protein
NAFLD: Non-alcoholic fatty liver disease
p-HSL: Hormone-sensitive lipase phosphorylation
PLTP: Phospholipid transfer protein
PM: Plasma membrane
RCT: Reverse cholesterol transport
SAA: Serum amyloid A
SR-BI: Scavenger receptor class B type 1
TNF-α: Tumor necrosis factor-α
UCP1: Uncoupling protein 1
VLDL: Very low density lipoprotein
WAT: White adipose tissue
